# Immune Checkpoint Blockade Therapy for Advanced Cutaneous Squamous Cell Carcinoma in Immunosuppressed Patients, Transplant Recipients, and Individuals with Hereditary Syndromes: A Narrative Review

**DOI:** 10.3390/cancers17223681

**Published:** 2025-11-17

**Authors:** Marta Pabianek, Aleksandra Lesiak, Joanna Narbutt, Branka Marinovic, Magdalena Ciazynska

**Affiliations:** 1 Chemotherapy Unit and One-Day Chemotherapy Unit, Specialist Oncology Hospital NU-MED, 97-200 Tomaszów Mazowiecki, Poland; 2 Department of Dermatology, Paediatric Dermatology and Oncology Clinic, Medical University of Łódź, 91-347 Łódź, Poland; 3 Laboratory of Autoinflammatory, Genetic and Rare Skin Disorders, Medical University of Łódź, 91-347 Łódź, Poland; 4 Department of Dermatology and Venereology, University Hospital Center Zagreb, School of Medicine, University of Zagreb, 10000 Zagreb, Croatia

**Keywords:** squamous cell carcinoma, immunotherapy, treatment challenges

## Abstract

Cutaneous squamous cell carcinoma (cSCC) is the second most common skin cancer, with incidence steadily increasing due to cumulative ultraviolet (UV) exposure, impaired immune surveillance, and chronic tissue damage. While most cases are effectively managed with surgical excision, a subset progress to locally advanced or metastatic disease, associated with high recurrence rates, limited curative options, and poor prognosis. The introduction of immune checkpoint inhibitors (ICIs) targeting the PD-1/PD-L1 axis has significantly altered the management of advanced cSCC. Cemiplimab and pembrolizumab are now established systemic therapies, producing durable responses in a proportion of patients. These outcomes reflect the typically high tumor mutational burden and immunogenic microenvironment of cSCC. However, therapeutic decision-making remains particularly complex in several high-risk populations, including solid organ transplant recipients at risk of allograft rejection, patients with chronic dermatologic disorders or non-healing wounds that predispose to carcinogenesis, and individuals with rare hereditary syndromes such as recessive dystrophic epidermolysis bullosa. These so-called challenging populations are frequently excluded from pivotal trials, resulting in limited evidence regarding efficacy, safety, and optimal treatment strategies. This review summarizes current evidence on the management of advanced cSCC in high-risk and underserved patient groups, integrating trial data, real-world evidence, and contemporary guidelines. It also highlights key gaps in knowledge and outlines future directions, with particular focus on the interplay between host immune status, tumor biology, and therapeutic response.

## Simple Summary

Cutaneous squamous cell carcinoma is one of the most common types of skin cancer and is usually treated successfully with surgery. However, in some patients, the disease spreads or becomes too advanced for surgery, leading to serious health risks and limited treatment options. In recent years, new drugs that help the immune system recognize and attack cancer cells have shown promising and long-lasting results. Unfortunately, people with weakened immune systems, chronic skin damage, or rare genetic conditions are often excluded from major clinical trials, leaving doctors uncertain about how best to treat them. This review aims to gather and explain what is currently known about treating advanced squamous cell carcinoma in these high-risk groups. The findings may help researchers and clinicians better understand how the immune system and tumor biology influence treatment response, and guide future studies to improve care for these vulnerable patients.

## 1. Introduction

Cutaneous squamous cell carcinoma (cSCC) is a major public health concern and represents the second most frequent form of nonmelanoma skin cancer after basal cell carcinoma [1]. While historically considered less common than basal cell carcinoma, recent data from multiple regions suggest that its incidence is approaching similar levels, reflecting both improved detection and changing demographic patterns [2,3]. Current global estimates indicate a continued year-on-year rise in new diagnoses, largely attributable to cumulative ultraviolet (UV) radiation exposure, increased longevity, a higher prevalence of immunosuppressive conditions or therapies, and the expansion of dermatologic screening programs [4].

From a clinical perspective, cSCC is notable for its broad biological spectrum. Most tumors are detected at an early stage and are amenable to curative local treatment, resulting in five-year survival rates exceeding 90% [5,6]. Nevertheless, a subset displays aggressive behavior, characterized by deep local invasion, recurrence, and metastatic spread, particularly to regional lymph nodes. While distant metastases remain relatively uncommon, they are associated with significant morbidity and mortality. In certain geographic areas, mortality rates from cSCC approach those reported for cutaneous melanoma [4,7]. While most cases present as localized disease and can be cured with surgical excision, a minority of patients develop advanced disease—either locally advanced cSCC (la-cSCC) or metastatic cSCC (m-cSCC)—which carries a significantly worse prognosis and presents complex therapeutic challenges.

In patients with locally advanced or metastatic cSCC who are not suitable for curative surgery or radiotherapy, systemic therapy is the standard of care, with immune checkpoint inhibitors (ICI) targeting PD-1 representing the preferred option. Cemiplimab was the first PD-1 (programmed cell death protein 1) inhibitor approved in this setting, based on the phase II EMPOWER-CSCC-1 trial, which demonstrated an objective response rate (ORR) of 47.2% and a median progression-free survival (PFS) of 26 months [8]. Pembrolizumab, evaluated in the phase II KEYNOTE-629 trial, achieved an ORR of 34.3% in patients with recurrent/metastatic cSCC and 50.9% in those with locally advanced disease; median duration of response was not reached in either cohort, indicating that more than half of the responders maintained their response at the time of analysis, and highlighting the potential for durable benefit [9].

However, it should be emphasized that both EMPOWER-CSCC-1 and KEYNOTE-629 did not include patients from so-called challenging populations. This category encompasses individuals whose clinical profile makes treatment particularly difficult and outcomes generally poorer—for example, those with marked immunosuppression (such as recipients of solid organ transplants, patients with hematologic cancers, or uncontrolled HIV), individuals with serious coexisting illnesses that limit therapeutic options, and cases with unusual tumor sites or uncommon metastatic patterns. Consequently, the results of these landmark trials may not fully capture the realities seen in everyday clinical practice, where such high-risk patients are encountered more frequently and often require highly tailored treatment approaches.

Therefore, this review aims to provide a comprehensive and focused synthesis of current evidence regarding the use of immune checkpoint blockade in advanced cutaneous squamous cell carcinoma, particularly among high-risk and underserved populations. Specifically, we seek to summarize the available data on the efficacy and safety of PD-1 inhibitors in immunosuppressed patients, transplant recipients, and individuals with hereditary syndromes; discuss the biological and immunological mechanisms underlying treatment response and resistance in these groups; and identify existing knowledge gaps and outline key directions for future research.

## 2. Materials and Methods

A search was conducted in PubMed using “immune checkpoint blockade”, and (“scc” or “squamous cell carcinoma”), and terms for high-risk group patients such as “immunosuppressed”, or “transplant”, or “dystrophic epidermolysis bullosa”, or “xeroderma pigmentosum” as keywords. Only English language studies were included in the narrative review. Studies from 2022 onward were considered as a time limit. We identified a total of 243 studies. Only studies, including clinical trials, observational studies, or metanalyses, reporting immune checkpoint blockade therapy for advanced cutaneous squamous cell carcinoma were included. Studies on safety or adverse effects were not included in this narrative review. We decided to include a total of 46 studies that met the inclusion criteria. We reported studies, even with preliminary data from clinical studies, that were published from 2022 onwards, while ongoing clinical trials with no published data were not included in this review. The purpose is to summarize the latest evidence produced and reviewed regarding immune checkpoint blockade therapy for advanced cutaneous squamous cell carcinoma in immunosuppressed patients, transplant recipients, and individuals with hereditary syndromes.

## 3. High-Risk Populations Predisposed to Advanced cSCC

The development of cutaneous squamous cell carcinoma is the result of a complex interplay between environmental, immunological, and genetic determinants [6,10]. Although less frequently diagnosed in individuals with darker skin phototypes, mortality rates in this group are markedly higher—18.4% compared with approximately 4% in the general population [4,11].

Immunosuppression is a well-recognized and significant risk factor for the development of cutaneous squamous cell carcinoma. Solid organ transplant recipients face an exceptionally high risk—up to 200 times greater than that of the general population [12]. Similarly, individuals with chronic lymphocytic leukemia have a 3.66-fold increased risk, while those living with HIV show a 2.76-fold higher incidence [13,14]. Other groups at elevated risk include hematopoietic stem cell transplant recipients, patients with other hematologic malignancies, and individuals with chronic autoimmune diseases. Managing cSCC in immunosuppressed patients presents distinct clinical challenges. Immunosuppression is linked to more aggressive tumor biology and poorer survival outcomes. In this setting, cSCC is more likely to be poorly differentiated, multifocal, recurrent, and metastatic [13,15,16,17]. Disease-specific survival is significantly worse compared with immunocompetent patients, with more than a twofold increase in the risk of death from cSCC [15]. These patients frequently have multiple comorbidities, and due to the aggressive nature of the disease, achieving a cure often requires more intensive treatment [13]. This necessity can create a delicate balance between complete tumor clearance and the preservation of function.

Also, chronic skin injuries—including non-healing wounds, longstanding scars, burns, ulcers, and sinus tracts—are recognized risk factors for the development of high risk cSCC. In such settings, carcinogenesis is thought to be driven by prolonged inflammation: repeated cycles of tissue injury and repair, along with sustained exposure to pro-inflammatory mediators, gradually create local conditions that favor malignant change. A well-known example is the Marjolin’s ulcer, where keratinocytes in scarred or chronically damaged skin progressively accumulate genetic alterations, often because of oxidative stress, persistent DNA damage, and reduced local immune surveillance [18,19,20]. Marjolin’s ulcers are frequently misinterpreted as chronically infected wounds, persistent scars, or ulcerations. Certain clinical features should raise suspicion for malignant transformation, including foul odor, pain accompanied by purulent or bloody discharge, failure to heal despite appropriate care, and the presence of a dense vascular network within the lesion. The malignant cells found in Marjolin’s ulcers tend to be particularly aggressive, with a higher metastatic potential compared to many other cutaneous malignancies. Spread to regional lymph nodes is most common, but distant metastases to the brain, liver, lungs, and kidneys have also been documented in the literature [18]. Reported latency periods between the initial insult and tumor onset are long—frequently measured in years or even decades—which highlights the need for ongoing clinical observation in patients with chronic skin lesions [21].

Genetic predisposition plays a central role in certain high-risk populations. Inherited disorders associated with defective DNA repair or skin fragility—such as xeroderma pigmentosum, oculocutaneous albinism, Kindler syndrome, recessive dystrophic epidermolysis bullosa, Fanconi anemia, and Lynch syndrome/Muir–Torre syndrome—carry a markedly elevated lifetime risk of cSCC, often with earlier onset and more aggressive disease course. Patients with dystrophic epidermolysis bullosa, for example, may develop multiple aggressive tumors by early adulthood, particularly at sites of chronic trauma [22].

Other dermatological and systemic conditions further predispose to cSCC. Cutaneous squamous cell carcinoma that develops in areas affected by chronic inflammatory conditions, such as hidradenitis suppurativa, lichen planus, scar tissue after radiotherapy, around ostomy sites, represents a particularly complex therapeutic challenge [23]. This is due to several overlapping factors. First, diagnosis in these patients is often delayed, which increases the likelihood of encountering locally advanced tumors at presentation. Second, the malignancy arises in skin already compromised by the underlying disease, which predisposes to postoperative complications such as wound dehiscence. This tissue is frequently less elastic, fibrotic, and more difficult to close or reconstruct with flaps, and it is often unsuitable for further irradiation. Chronic inflammation in these areas can act as a so-called “field of cancerization,” meaning that recurrences may occur even after appropriate treatment of the primary tumor. In the case of hidradenitis suppurativa, the presence of fistulous tracts provides an ideal pathway for tumor extension, often resulting in more extensive local spread than initially expected and complicating complete excision. These tracts can run deep and involve anorectal or urogenital structures.

## 4. Immunotherapy, Immunosuppression and Advanced cSCC

Patients with reduced immune function represent one of the main groups at increased risk of advanced cSCC. In immunosuppressed patients, including solid organ transplant recipients, individuals with hematologic malignancies, and those living with HIV, cutaneous squamous cell carcinoma (cSCC) often demonstrates more aggressive behavior, higher recurrence rates, and greater metastatic potential [6,13,14,24]. The duration and intensity of immunosuppressive therapy further influence tumor development, and certain agents such as azathioprine, cyclosporine, and mycophenolate mofetil have documented procarcinogenic effects, partly through UV-sensitizing mechanisms [6].

Chronic immunosuppression profoundly reshapes the tumor microenvironment, facilitating immune evasion and promoting tumor progression [6,13,24]. Sustained inhibition of immune effector pathways results in attenuated antigen presentation, diminished infiltration of cytotoxic CD8^+^ T lymphocytes, and the expansion of regulatory T cells and myeloid-derived suppressor cells, which collectively establish an immunosuppressive milieu [13,24]. In addition, prolonged exposure to immunosuppressive agents may modulate the expression of immune checkpoint molecules, including PD-L1, on tumor and stromal cells, thereby impairing T-cell activation and promoting functional exhaustion [24]. These converging mechanisms undermine antitumor immune surveillance and contribute to the emergence of highly aggressive cutaneous squamous cell carcinoma (cSCC) phenotypes [6,13,14,15,16,24]. Although immunotherapy has shown encouraging results in immunocompetent patients, its safety and effectiveness in individuals with compromised immune systems remain far less explored. It is important to emphasize that the currently available evidence varies considerably in methodological quality. Most published data concerning immunosuppressed or transplant recipients originate from individual case reports or small case series, while only a few prospective or multicenter studies have been conducted. Therefore, the conclusions drawn from these observations should be interpreted with caution, as they primarily reflect anecdotal experience rather than high-level, randomized evidence. A recent phase I trial evaluating cemiplimab in kidney transplant recipients with advanced cSCC reported a response rate of 46%, with none of the 12 participants experiencing graft rejection [25]. In addition, several case reports published in recent years have described successful use of immunotherapy for advanced cSCC in solid organ transplant recipients [26,27]. Early findings from the C.A.S.E. study, which assessed cemiplimab in this high-risk group, indicated that 47% of 19 immunosuppressed patients achieved either a complete or partial response, while one patient developed transplant rejection [28]. At the 2025 ASCO Annual Meeting, interim findings from the phase IV CASE study (NCT03836105) were presented, providing real-world data on cemiplimab in patients with locally advanced or metastatic cutaneous squamous cell carcinoma (laCSCC/mCSCC). The analysis included 254 patients treated across 65 US academic and community centers, of whom 17% were immunocompromised. All patients received at least one dose of cemiplimab, with a median treatment duration of 35 weeks. The investigator-assessed overall response rate (ORR) was 43.7% in the total study population and 55.2% among those with available response evaluations, with complete responses observed in 15.7% and 19.9% of patients, respectively. Among the 17% of patients who were immunocompromised, detailed efficacy data were not reported; however, the safety profile was consistent with that of the overall study population. Median progression-free survival (PFS) was 14.7 months, and the estimated 12-month survival rate reached 59.5%. Immune-related adverse events occurred in 29.9% of participants, while treatment-related serious adverse events were reported in 7.5%. The safety profile was consistent with prior clinical trial data. These real-world results support the robust antitumor activity and manageable safety profile of cemiplimab, aligning closely with the outcomes of the pivotal EMPOWER-CSCC-1 trial [29].

Although the available evidence remains limited, emerging data provide insights into strategies for adjusting immunosuppressive regimens during immune checkpoint inhibitor (ICI) therapy in solid organ transplant recipients (SOTRs) to reduce the risk of rejection without compromising antitumor efficacy. Rejection events are thought to be driven by alloreactive CD8+ T cells, whose activation by ICI may vary depending on the type, intensity, and duration of baseline immunosuppression [30,31]. The influence of different immunosuppressive regimens on immune checkpoint inhibitor (ICI) efficacy and rejection risk remains an important area of ongoing research. Calcineurin inhibitors inhibit IL-2–dependent T-cell activation and may reduce ICI antitumor activity while helping to maintain graft tolerance. In contrast, mTOR-based regimens have antiproliferative and potential antineoplastic effects and, in observational studies—especially among kidney transplant recipients—have been associated with lower rates of cutaneous squamous cell carcinoma and favorable graft outcomes. Further research should clarify how these agents modulate T-cell function and checkpoint signaling to optimize immunosuppressive protocols that balance antitumor efficacy with graft preservation in immunocompromised patients. Early case reports described clinical benefit from ICI in SOTRs who continued their immunosuppressive therapy, although episodes of allograft rejection were also reported [32,33]. One widely cited case detailed a patient achieving tumor control with ICI while receiving a novel regimen combining an mTOR inhibitor with dynamic glucocorticoid dosing [34]. Table 1 summarizes studies evaluating non-surgical approaches to the treatment of cutaneous squamous cell carcinoma in immunosuppressed patients.

Three other prospective studies have evaluated the safety and efficacy of ICI in kidney transplant recipients (KTRs) with cutaneous squamous cell carcinoma (CSCC) and other malignancies. The first, a multicenter phase I trial conducted in Australia, enrolled 17 KTRs with metastatic solid tumors treated with nivolumab [42]. Participants remained on their pre-trial immunosuppressive protocols, most receiving two-drug (53%) or three-drug (41%) regimens, with prednisone being the most common agent (76%). Over a median follow-up of 28 months, no cases of irreversible allograft rejection occurred, although none of the six CSCC patients achieved a response.

The second trial tested a standardized low-dose tacrolimus plus low-dose prednisone regimen in eight KTRs with advanced melanoma or non-melanoma skin cancers receiving nivolumab [35]. The composite primary endpoint was overall response rate (ORR) without graft loss at 4 months. No patient achieved an objective response, and one experienced treatment-related allograft loss (TRAL). Patients with progressive disease could escalate to combination therapy with ipilimumab and nivolumab. Of six such patients, two achieved complete responses (one with TRAL), while four had progression (one with TRAL). Analysis of PD-L1 expression and rejected graft specimens showed high PD-L1 levels and activated T-cell infiltrates, underscoring the role of the PD-1/PD-L1 axis and T-cell–mediated immunity in allograft rejection.

CONTRAC Study, in 12 KTRs with advanced CSCC, assessing cemiplimab (anti–PD-1) given alongside a structured immunosuppression protocol known as the Harvard regimen [25]. This approach involved moderate-dose prednisone administered in a mini-pulse schedule (40 mg daily for four days beginning the day before infusion, tapered to 20 mg for three days and then 10 mg daily) in combination with continuous therapeutic-level mTOR inhibition (target trough 4–6 ng/mL). At a median follow-up of 6.8 months, there were no rejection episodes or graft losses, and the ORR was 46% among 11 evaluable patients. Collectively, these trials highlight both the potential and the complexity of using ICIs in KTRs with CSCC, demonstrating that careful adjustment of immunosuppressive therapy—particularly through mTOR-based regimens—may enable meaningful antitumor responses while mitigating the risk of graft rejection [25,43].

However, not all immunotherapy strategies have yielded similarly positive outcomes. In a phase I/II trial investigating a combination of nivolumab, tacrolimus, and prednisone—with or without ipilimumab—in kidney transplant recipients with advanced skin cancers, no tumor regression was observed, and three of the eight patients lost their grafts because of treatment [35]. A multicenter retrospective analysis also found that among kidney transplant patients treated with immune checkpoint inhibitors for cSCC, the overall response rate was 33.3%, while the incidence of acute rejection reached 37.5% [44].

In patients with concurrent hematologic malignancies, nivolumab demonstrated activity against locally advanced and metastatic cSCC, with no significant difference in treatment-related adverse events compared to immunocompetent individuals [45]. Conversely, another retrospective study reported that immunotherapy was notably less effective in this subgroup than in patients without immune impairment [46].

For people living with HIV (PLWH), published evidence is limited mostly to case reports, many of which have shown favorable outcomes [36,37]. A multi-institutional phase I study found that nivolumab had a comparable safety profile and efficacy in PLWH as in immunocompetent patients, and its use in Kaposi sarcoma and other solid tumors did not significantly affect HIV viral load or CD4 counts [43]. Further research in other cancers and with different immunotherapy agents supports both the safety and antitumor activity of these drugs in PLWH [43,47,48]. While these findings may extend to cSCC, more dedicated studies are required.

## 5. Immunotherapy in cSCC Arising in Chronically Damaged Skin (Marjolin’s Ulcer)

Another important clinical context involves tumors arising in areas of long-standing wounds, scars, or chronic inflammation. The mechanisms underlying cSCC arising in chronically damaged skin have been the subject of debate for more than a century. Multiple etiological factors may drive malignant transformation, including chronic scar tissue, which tends to have a reduced presence of immune cells, thereby facilitating evasion from immune surveillance [19,49].

In a retrospective study by Miodovnik et al., the clinical outcomes of 84 patients with cutaneous squamous cell carcinoma (cSCC) treated with anti–PD-1 immunotherapy (cemiplimab or pembrolizumab) at a single tertiary referral center were analyzed. Among these, 9 patients (11%) had tumors arising within chronic ulcers, commonly referred to as Marjolin’s ulcers. Six out of these nine lesions (67%) were located on the lower extremities, areas with minimal sun exposure [50,51].

Treatment efficacy in this subgroup was notably lower compared to the remaining patients. In ulcer-related cSCC, only 2 patients (22%) achieved a partial response (PR), and none achieved a complete response (CR). In contrast, among the 75 patients without ulcer-associated cSCC, the PR rate was 53% (40 patients), and the CR rate was 26% (20 patients). This difference was statistically significant (*p* < 0.001). Progression-free survival (PFS) was also significantly shorter in ulcer-related cases. The median PFS in this group was only 2.1 months, whereas in the non-ulcer group it was not reached (*p* < 0.001). Median overall survival (OS) was 17 months in the ulcer group versus 30 months in the non-ulcer group (*p* = 0.02), although this did not reach formal statistical significance. In multivariate analysis, ulcer-associated cSCC was identified as an independent risk factor for disease progression, with a hazard ratio (HR) of 7.56 (95% CI: 1.26–40.74; *p* = 0.027) [50,51].

In this study Miodovnik et al. reported that 89% (67 of 75) of non-ulcerated cutaneous squamous cell carcinomas (cSCC) occurred in sun-exposed areas. In contrast, most SCC associated with chronic ulcers (SCC-MU) were located below the umbilicus (6 of 9 cases, 67%), a region less commonly subjected to solar damage. Typically, cSCC exhibit a high tumor mutational burden (TMB) as a result of cumulative ultraviolet (UV) radiation exposure and subsequent DNA injury. The lower response rates observed in MU cases may be linked to a reduced mutational load stemming from a non–UV-related pathogenesis. In line with this, Shalhout et al. described a series of five patients with MU-SCC treated with PD-1 inhibitors [52].

In a retrospective analysis conducted at Massachusetts General Hospital between 2016 and 2020, Shalhout et al. reviewed cases of patients with MU treated with anti–PD-1 agents [52]. They described a case series of five patients with MU-SCC who received PD-1 blockade, including two treated with pembrolizumab and three with cemiplimab. Four patients initiated immunotherapy as first-line treatment. One case demonstrated durable disease control for 12 months during therapy, with continued stability after discontinuation. Among the four patients who experienced progression, two achieved disease control at the 5-month mark. At 12 months following the start of anti–PD-1 treatment, overall survival was 60% (three patients) in this small cohort. The authors also provided detailed descriptions of the clinicopathologic characteristics and treatment outcomes for these MU-SCC cases [52].

Several other biological mechanisms may explain these unfavorable outcomes and reduced responsiveness to PD-1 blockade in patients with cSCC arising in chronically damaged skin [53]. Firstly, cSCC developing in chronic ulcers may exhibit a distinct and more aggressive tumor phenotype with higher invasive and metastatic potential. Secondly, diagnosis in these cases is often delayed, as malignant changes can be masked by the chronic inflammatory and granulation tissue seen in non-healing wounds. Persistent tissue injury and inflammation reshape the tumor microenvironment toward an immune-tolerant state. Repeated cycles of damage and repair activate fibroblasts and promote angiogenesis, accompanied by secretion of immunomodulatory cytokines such as IL-6, IL-10, and TGF-β. These alterations impair antigen presentation and cytotoxic T-cell infiltration, creating a protumorigenic niche that facilitates immune evasion and may contribute to reduced responsiveness to PD-1 blockade in ulcer-related cSCC [19,49,53,54,55].

Another important factor may be local iron overload in the tissue surrounding chronic venous ulcers [54]. X-ray spectrometry studies have demonstrated significantly elevated iron concentrations in the skin adjacent to such ulcers. This phenomenon is attributed to erythrocyte extravasation due to chronic venous hypertension, breakdown of red blood cells, and deposition of hemosiderin in the dermis and subcutaneous tissue. Ferric iron ions can promote oxidative stress, generate free radicals, and activate matrix metalloproteinases, leading to chronic tissue damage and impaired wound healing [54,55]. Growing evidence suggests that chronic iron deposition may actively contribute to carcinogenesis by inducing DNA damage, genomic instability, and protein and membrane injury, while fostering a pro-tumorigenic microenvironment that supports cancer cell proliferation, migration, and metastasis.

Preclinical studies have shown that iron accumulation can drive metabolic changes in tumor cells toward a more aggressive phenotype. Furthermore, iron overload may disrupt the balance and function of T-lymphocyte subsets, alter surface marker expression, and impair their ability to mount effective antitumor immune responses. Lymphocytes, with their limited capacity for iron storage, may be particularly vulnerable to oxidative damage in iron-rich environments [54,55,56,57].

The literature on immunotherapy for cSCC arising in chronic wounds or scars remains limited almost entirely to small retrospective analyses and individual case reports. While these publications offer valuable clinical insights, they should be regarded as hypothesis-generating rather than confirmatory evidence.

Persistent tissue injury and inflammation create a distinct tumor environment, but some patients face additional risk due to inherited genetic disorders. The following section addresses these hereditary conditions.

## 6. Immunotherapy in cSCC Developing in Patient with Certain Hereditary Syndromes

Cutaneous squamous cell carcinoma (cSCC) may develop in the context of certain hereditary syndromes that predispose patients to early-onset and often aggressive skin cancers. These syndromes frequently involve defects in DNA repair, genomic stability, or immune regulation, creating a unique biological background that can influence both tumor behavior and therapeutic response. These inherited disorders not only predispose to exceptionally aggressive forms of cSCC but also create unique biological settings that influence tumour–immune interactions and responsiveness to immunotherapy.

Beyond clinical variability, hereditary syndromes also shape distinct tumour–immune microenvironments. In recessive dystrophic epidermolysis bullosa (RDEB), chronic wounding, fibrosis, and persistent TGF-β1 activation foster an immunosuppressive and cytokine-rich milieu that supports tumour progression and limits cytotoxic T-cell function [58,59,60]. Conversely, in xeroderma pigmentosum (XP), an extremely high mutational burden and neoantigen load theoretically enhance tumour immunogenicity [61]; however, chronic UV-induced inflammation and PD-L1 upregulation may counterbalance this effect, leading to heterogeneous responses to immune checkpoint inhibition [62,63,64].

Due to the extreme rarity of hereditary syndromes such as recessive dystrophic epidermolysis bullosa (RDEB) and xeroderma pigmentosum (XP), most available data derive from isolated case reports or very small patient cohorts. Consequently, the therapeutic outcomes summarized in this review should be interpreted as anecdotal evidence reflecting real-world clinical experience rather than high-level scientific proof.

Recessive dystrophic epidermolysis bullosa (RDEB) is an uncommon inherited mechanobullous condition marked by fragile skin that blisters easily, chronic wounds often colonized by bacteria, and a markedly elevated risk of developing cutaneous squamous cell carcinoma (cSCC), which tends to metastasize early and frequently follows a severe, potentially fatal course [58,65,66,67,68,69,70]. RDEB is caused by biallelic pathogenic variants in the COL7A1 gene, which encodes type VII collagen, a key component of anchoring fibrils that attach the epidermis to the dermis. Loss or dysfunction of type VII collagen results in dermo-epidermal separation, chronic skin fragility, and impaired wound healing. In RDEB, cSCC typically arises in areas of chronic injury accompanied by persistent inflammation, with innate immune recognition of microbial products playing a key role as a risk factor [59,71]. Endogenous mutational mechanisms, primarily driven by apolipoprotein B mRNA editing enzyme, catalytic polypeptide-like (APOBEC), result in the rapid accumulation of a substantial mutational load in the skin and tumors of affected individuals [72]. Additional contributors to the aggressive behavior of cSCC in RDEB include scarring, fibroblast activation, upregulated transforming growth factor beta-1 (TGF-β1) production, and other tissue repair pathways [60]. Because chronic non-healing wounds represent a major driver of carcinogenesis in RDEB, therapeutic strategies aimed at restoring COL7A1 function—such as gene or cell-based therapies—may improve wound closure, reduce persistent inflammation, and thereby decrease long-term cSCC risk.

Although inflammation is thought to be a critical factor in RDEB pathogenesis, the immune microenvironment of RDEB-related cSCC has yet to be fully characterized. Despite cSCC being the leading cause of mortality in patients with RDEB, there is limited evidence regarding adjuvant, neoadjuvant, or palliative therapeutic approaches in this setting [68], with the literature consisting mainly of isolated case descriptions [73,74,75,76,77].

Despite the scarcity of cases, several reports have begun to explore systemic PD-1 inhibitor therapy for cSCC in RDEB. In a systematic review encompassing 157 RDEB–cSCC cases, only a handful were treated with PD 1 inhibitors—cemiplimab (8 cases), nivolumab (1 case), and pembrolizumab (2 cases). One patient received pembrolizumab in combination with intralesional administration of the oncolytic viral therapy talimogene laherparepvec (T-VEC). Among the individuals treated with cemiplimab, two achieved a complete response, while three maintained stable disease [77,78,79,80,81]. The outcome for a sixth, more recent patient in this group has not yet been reported [69]. Cemiplimab therapy was generally well tolerated, with the most frequently observed side effects being mild fatigue and nausea. In the single reported case involving nivolumab, the treatment course was also well tolerated aside from fatigue; notably, the patient remained in remission for four months after discontinuation of therapy [82].

Of the two patients managed with pembrolizumab, one experienced more than a 50% reduction in the size of cSCC lesions along with complete resolution of ulcerations after 12 months of treatment. The other patient, however, succumbed to disease progression [75,76]. Immune-related thyroiditis was the only treatment-associated adverse event documented with pembrolizumab [76]. For the solitary case in which T-VEC was used, injections were delivered directly into metastatic lesions in the cervical and axillary lymph nodes. Unfortunately, tumor progression occurred, and the patient died five months later [75]. Table 2 provides a summary of reported cSCC cases treated with immunotherapy.

Xeroderma pigmentosum (XP) is a rare autosomal recessive geno-photodermatosis caused by defects in the DNA repair machinery, resulting in marked hypersensitivity to ultraviolet (UV) radiation. Individuals with XP are predisposed to severe sunburn, xerosis, progressive pigmentary changes, accelerated photoaging, and a markedly increased risk of developing malignant skin tumors in chronically sun-exposed regions, such as the face, neck, and scalp [83,84,85] The lifetime risk of non-melanoma skin cancers, including basal cell carcinoma and cSCC, is estimated to be up to 10,000-fold greater than in the general population. When skin cancers develop in XP, they often behave more aggressively, with a higher likelihood of metastasis and cancer-related mortality. The median life expectancy is around 32 years, and approximately 60% of patients die before reaching the age of 20 [86,87,88]. Table 3 presents anti-PD-1 treatment experiences in xeroderma pigmentosum patients and the associated outcomes.

Molecular analyses have shown that skin cancers in XP harbor, on average, a 3.6-fold higher mutational burden than their sporadic counterparts. This is especially pronounced in XP subtypes with defects in global genome nucleotide excision repair (NER), such as XP-C and XP-E, as well as in those lacking functional translesion synthesis polymerase η, as in XP-V. In certain subgroups, the mean tumor mutational burden (TMB) for single-base substitutions may reach 350 mutations per megabase. The most common mutation type in XP-related tumors is the C>T transition, particularly at dipyrimidine sequences, which are characteristic signatures of UV-induced DNA damage. Distinct mutational profiles are observed across XP subtypes, reflecting differences in DNA repair capacity and cumulative UV exposure history.

Given this high mutational load, cSCC in XP patients could, in theory, be highly responsive to immune checkpoint blockade. While cemiplimab and other immune checkpoint inhibitors (ICIs) have demonstrated substantial improvements in progression-free and overall survival for immunocompetent patients with advanced cSCC [61,89,90,91,92,93,94,95,96,97,98,99,100,101,102,103,104], evidence for their efficacy in XP remains limited.

Certain hereditary syndromes, such as Fanconi anemia, Bloom syndrome, and Li-Fraumeni syndrome, are also associated with an increased risk of developing skin cancers, including cutaneous squamous cell carcinoma (cSCC). These conditions are characterized by genomic instability and defects in DNA damage response mechanisms. Although evidence on the use of immune checkpoint inhibitors in these rare syndromes remains very limited, their underlying biology suggests potential susceptibility to immunotherapy, particularly in tumors with high mutational burden or impaired DNA repair. Future studies and case reports evaluating the efficacy and safety of such treatments in genetically predisposed populations could provide valuable insights for precision oncology. Taken together, these examples show how immune function, tissue integrity, and genetic background interact to shape the behavior of advanced cSCC.

These three high-risk patient populations—immunosuppressed individuals (including solid organ transplant recipients), patients with chronic wound–associated or inflammation-driven cSCC, and those with hereditary cancer-predisposition syndromes—share overlapping but distinct immunological mechanisms that influence disease aggressiveness and therapeutic response. A comparative overview of these mechanisms, their clinical implications, and emerging management strategies is summarized in Table 4.

## 7. Emerging Biomarkers and Molecular Predictors

Recent studies have identified several candidate biomarkers that may refine patient selection for immunotherapy in advanced cSCC. Tumor mutational burden (TMB) and UV-signature mutations are associated with tumor immunogenicity and may correlate with better response to PD-1 blockade, although standardized thresholds for cSCC are still lacking [22,91,92,104]. Conversely, tumors arising in non–UV-related contexts, such as Marjolin’s ulcers or RDEB-associated cSCC, often show lower TMB and distinct mutational patterns, which may contribute to reduced responsiveness [50,51,52,60,72]. Exploratory data also link PD-L1 expression and the density of tumor-infiltrating lymphocytes with treatment outcomes, but their predictive value remains inconsistent [41]. Genomic alterations in genes such as NOTCH1/2, TP53, and FAT1 appear to shape the tumor–immune microenvironment, though they are not validated predictive markers [22,60]. Further molecular and immunologic profiling—including transcriptomic analyses and circulating tumor DNA approaches—may, in the future, support precision management and real-time disease monitoring in these complex patient populations.

## 8. Conclusions

The management of advanced cutaneous squamous cell carcinoma (cSCC) in high-risk populations remains one of the most complex challenges in dermatologic oncology. Although PD-1 inhibitors such as cemiplimab and pembrolizumab have improved outcomes in the general population, the pivotal trial data do not adequately reflect the reality of patients with profound immunosuppression, significant comorbidity, or biologically atypical tumors. For these individuals, every therapeutic decision requires careful individualization and ideally input from a multidisciplinary team.

In transplant recipients, clinical experience suggests that adjusting baseline immunosuppression with mTOR-based regimens and short peri-infusion corticosteroid courses may help balance rejection risk with anticancer efficacy. For tumors arising in chronic wounds or scar tissue, responses to immunotherapy are often less robust, potentially due to reduced mutational burden and a pro-tumorigenic microenvironment. Patients with rare hereditary cancer-predisposition syndromes, such as recessive dystrophic epidermolysis bullosa (RDEB) or xeroderma pigmentosum, represent another underserved group. Although evidence remains limited, durable responses have been reported, particularly in tumors with high mutational loads, underscoring the therapeutic potential of PD-1 blockade in carefully selected cases.

However, translating these advances into real-world practice poses substantial ethical and practical challenges. The high cost of immune checkpoint inhibitors and the unequal access to advanced oncologic therapies across regions and healthcare systems may limit their availability for patients with rare or complex diseases. Moreover, close monitoring for immune-related adverse events requires specialized, multidisciplinary care that may not be accessible in all clinical settings. Further ethical concerns arise from the scarcity of clinical trial data in these populations, underscoring the importance of equitable representation in future studies. Overcoming these barriers will be essential to ensure that therapeutic progress translates into tangible benefits for all patient groups, not only those represented in pivotal trials.

Moreover, this review synthesizes evidence of varying methodological rigor, spanning from anecdotal case reports to phase II trials and real-world studies. Although the inclusion of rare and high-risk populations provides valuable clinical context, the predominance of lower-level evidence inevitably limits the robustness of our conclusions. Accordingly, we acknowledge the need for further prospective, adequately powered studies to confirm the safety and efficacy of immune checkpoint inhibitors in these underserved patient populations.

Looking ahead, rational combinations of systemic PD-1 blockade with locoregional immunotherapies—such as intralesional oncolytic viral therapy (e.g., talimogene laherparepvec) or topical immunomodulators (e.g., imiquimod)—warrant prospective evaluation in advanced cSCC, particularly in immunocompromised and genetically predisposed patients. Case-level evidence suggests these approaches can augment local antigen release and presentation and might potentiate systemic responses, although outcomes have been variable and remain unproven. Carefully designed translational and clinical studies are needed to define efficacy, safety (including graft tolerance in SOTRs), optimal sequencing, and patient selection for such multimodal regimens [26,75,76].

Future progress will depend on prospective trials and translational research capable of dissecting the tumor–immune interactions unique to these settings. Equally important is the integration of preventive strategies, including regular dermatologic surveillance, rigorous photoprotection, chemoprevention in selected cases, and, in hereditary syndromes, emerging gene- and cell-based therapies. In RDEB, correction of the COL7A1 defect is particularly promising, as it may restore type VII collagen expression, improve wound healing, reduce the number of chronic non-healing ulcers, and thereby lower the long-term risk of cSCC. Looking ahead, a combined treatment paradigm that integrates surgery, immunotherapy, and gene therapy may become a realistic strategy—not only to control advanced disease but also to reduce recurrence and prevent secondary tumor development. Such integrative strategies will be essential to deliver durable benefit to patient groups who have long remained at the margins of clinical research.

Future investigations may focus on developing integrated therapeutic approaches that address both the molecular basis of disease and its clinical manifestations. In hereditary conditions such as RDEB, where chronic tissue damage drives carcinogenesis, the combination of gene repair strategies with immunotherapy could offer new opportunities for prevention and treatment. Exploring how restoration of skin integrity influences immune responses and tumor behavior may provide valuable insights for designing more effective, personalized interventions for these patients.

## Figures and Tables

**Table 1:** Summary of the studies investigating non-surgical treatment of cutaneous squamous cell carcinoma in immunosuppressed patients.

Study Design	Cohort	Treatment	Results	Author
Nonrandomized trial	12 renal transplant recipients	Cemiplimab	46% response rate to the treatment with no kidney rejection or loss	Hanna et al. [25]
Case study	1 heart transplant recipient	mTOR inhibitor prophylaxis + talimogene laherparepvec (T-VEC) injection	No allograft rejection occurred after treatment	Joo et al. [26]
Case study	1 renal transplant recipient	Cemiplimab	Complete disease remission with no allograft rejection after treatment	Ali et al. [27]
Prospective trial	12 renal transplant recipients	Nivolumab + tacrolimus + prednisone ± ipilimumab	Tacrolimus and prednisone failed to provide sufficient allograft protection	Schenk et al. [35]
Case study	1 HIV patient	Cemiplimab	Complete response with no toxicities	Alloghbi et al. [36]
Case study	1 AIDS patient	Cemiplimab-rwlc	No signs or symptoms of metastatic disease	Brereton et al. [37]
Case study	1 kidney transplant recipient	Pembrolizumab	No rejection, stable disease	Lakhani et al. (2021) [38]
Retrospective review study	2 kidney transplant recipients	Pembrolizumab	No rejection, progression disease	Tsung et al. (2021) [39]
Case Series	1 kidney transplant recipient	Pembrolizumab	Rejected, reacted pulse	Kumar et al. (2020) [40]
Retrospective review	1 kidney transplant recipient	Pembrolizumab	Rejected, progression disease	Venkatachalam et al. (2020) [41]

**Table 2:** Immunotherapy treatment of SCC in RDEB patients.

Age (yr)/Sex	Site(s) of SCC Under Treatment/ Tumor Size (cm)	Site(s) of Metastases	Treatment → Outcome	Adverse Events	Reference
33 F	Forearm	Axillary and infraclavicular lymph nodes, in-transit cutaneous and subcutaneous metastases on right upper limb	1. Exc → Metastasis 2. Cet → RC/RL + RC/RL + Metastasis 3. ECT + MTX → PR 4. Pem + T-VEC + Pan → Death from SCC	(Cet) Impaired wound healing; grade 2 allergic reaction with circulatory collapse, tightness in chest, erythema, fever, and chills	Medek et al. [75]
40 F	Forearm	Axillary and cervical lymph nodes; chest wall; pathologic fracture of left humerus due to metastatic SCC	1. Cet + RT → RC/RL 2. Amp → Metastasis 3. Niv → SD	(Cet) Impaired wound healing, lymphedema (Niv) Fatigue	Bruckner et al. [82]
32 M	Upper arm, > 5	Axillary lymph nodes	1. Res → RC/RL 2. 5-FU + MTX + Imi → Metastasis 3. Cem + RT → CR	Fatigue; nausea	Khaddour et al. [79]
28 F	Chest, 2–5	Subcutaneous SCC on right upper chest wall	1. Debulking surgery → PD 2. ECT → PD 3. Cem → PD	None	O’Sullivan et al. [78]
45 F	Head/neck; Lower leg; Foot	N/A	1. Exc → RC/RL 2. ECT → PR 3. Amp → PD 4. Pem → PR	(Pem) Immune-related thyroiditis	Piccerillo et al. [76]
51 F	Forearm, Hand, Knee	Inguinal lymph nodes	1. Amp → Metastasis 2. Pem → PD 3. Cet → SD	(Pem), development of new SCCs with reduced PD-L1 expression	Reimer et al. [73]
24 F	Unknown	N/A	1. Exc 2. Imi 3. Systemic retinoid 4. Cem Outcome unknown	N/A	Robertson et al. [69]
30 F	Back	N/A	1. Res → PD 2. MTX → PD 3. Cem → SD	Mild fatigue; worsening pruritus	Duong et al. [77]
34 M	Hand, > 5	N/A	1. Exc → PD 2. RT → PD 3. Cem → CR	Pruritus	Vasilev et al. [81]
16 M	Forearm, Upper leg, Foot	Inguinal lymph nodes	1. Exc → PD 2. Cem → PD 3. Amp → SD 4. Cet → PD	N/A	Trefzer et al. [80]
36 M	Head, Upper arm, Forearm, Hand, Foot, < 2	N/A	1. Exc → PD 2. Cem + RT → SD	N/A	Trefzer 2023 [80]

Amp: Amputation, Cem: Cemiplimab, Cet: Cetuximab, CHT: Unspecified chemotherapy, CR: Complete response, ECT: Electrochemotherapy, Erl: Erlotinib, Exc: Excision, 5-FU: 5-fluorouracil, Gem: Gemcitabine, Imi: Topical imiquimod 5%, Int: Intermediate RDEB, Meta: Metastases, MTX: Methotrexate, Niv: Nivolumab, NR: No response, Pan: Panitumumab, PD: Progressive disease, Pem: Pembrolizumab, PR: Partial response, Res: Resection, RC/RL: Local recurrence or relapse, RT: Radiotherapy, SD: Stable disease, Sev: Severe RDEB, T-VEC: Talimogene laherparepvec, N/A: Not available.

**Table 3:** Anti-PD1 drugs used in patients with XP and their outcomes.

Age	Sex	Treated Tumor	Localization	Treatment	Outcome	Non-Target Lesions	Reference
6 y	F	Sarcomatoid cSCC	Scalp	Nivolumab 3 mg/kg BW, 16 cycles in total	Complete remission	-	Chambon et al. [61]
6 y	M	Metastatic cSCC	Nose	Nivolumab, first pass with 6 cycles, second pass with 16 cycles	Complete remission	-	Sahin et al. [89]
7 y	F	Metastatic cSCC	left lower eyelid, right conjunctiva and cornea	Pembrolizumab 2 mg/kg BW, 9 cycles in total	Remission, except for cornea lesions finally treated with topical 5- fluorouracil	-	Steineck et al. [90]
7 y	M	Huge cSCC	the face with lymph nodes metastases of the neck	Cemiplimab initial 3 mg/kg BW, 24 cycles in total, therapy still ongoing	Complete remission		Gambichler et al. [62]
48 y	F	Metastatic SCC	Left tight with lymph node metastases	Pembrolizumab 2 mg/kg every 3 weeks	Partial response with regression of all metastases after 3 cycles	-	Deinlein et al. [63]
18 y	F	Unresectable SCC	Limbus of right eye	Pembrolizumab 2 mg/kg every 3 weeks	Complete regression after 8 months of therapy	Not response of BCCs on the face surgically removed	Ameri et al. [64]
19 y	M	Unresectable SCC	Right nasal cavity and orbit	Pembrolizumab 2 mg/kg every 3 weeks	Partial radiographic regression	-	Ameri et al. [64]
20 y	F	(i) Metastatic melanoma; (ii) unresectable SCC	(i) Unknown primary origin; (ii) maxillary sinus	(i) Ipilimumab 10 mg/kg every 3 weeks; (ii) Pembrolizumab 140 mg once a month	(i) Remarkable response; (ii) well response for 31 months until radiographic progression	Development of one BCC on right eyebrow treated with Mohs surgery	Ameri et al. [64]
7 y	F	Metastatic SCC	Right side of the face with, at first, the involvement of the right sphenoid bone, the cavernous sinus and the right carotid artery, and then the extension to surrounding tissues with lymph node metastases and leptomeningeal spread	Pembrolizumab 2 mg/kg every 3 weeks	A considerable decrease in tumor bulk and the resolution of leptomeningeal disease after five cycles; a long-term sustained stable disease	-	Steineck et al. [90]

**Table 4:** Overview of immunotherapy-related mechanisms, clinical challenges, and management strategies in high-risk populations with advanced cutaneous squamous cell carcinoma (cSCC).

High-Risk Population	Mechanisms (Pathogenic/Immune)	Clinical Challenges	Potential Strategies
Solid organ transplant recipients (SOTR)/immunosuppressed patients	Activation of alloreactive CD8⁺ T cells PD-L1 upregulation under chronic immunosuppression Reduced cytotoxic T-cell infiltration	Risk of graft rejection Reduced efficacy of immune checkpoint blockade under calcineurin inhibitors	Switch to mTOR-based immunosuppressive regimens Gradual steroid tapering Close graft monitoring (CONTACT study protocol)
Chronic wounds/Marjolin’s ulcer	Reduced tumor mutational burden (TMB↓) Pro-inflammatory and iron-rich microenvironment Impaired antigen presentation	Inferior and shorter response to immune checkpoint inhibitors Frequent local recurrence Limited surgical or radiotherapy options	Combine ICI with topical or locoregional immunotherapies (e.g., imiquimod, T-VEC) Optimize wound control and debridement Early biopsy of chronic ulcers
Hereditary syndromes (RDEB, XP)	COL7A1 defect and APOBEC-driven mutagenesis in RDEB Ultra-high tumor mutational burden in XP	Variable or suboptimal ICI efficacy High comorbidity burden Early-onset and aggressive disease course	Combine gene- or cell-based therapies (e.g., COL7A1 correction) with ICI Tailored systemic treatment Long-term dermatologic and oncologic surveillance

APOBEC—Apolipoprotein B mRNA Editing Catalytic Polypeptide-like enzymes, COL7A1—Collagen Type VII Alpha 1 Chain, ICI—Immune Checkpoint Inhibitor, mTOR—Mechanistic Target of Rapamycin, PD-L1—Programmed Death-Ligand 1, RDEB—Recessive Dystrophic Epidermolysis Bullosa, SOTR—Solid Organ Transplant Recipient, TMB—Tumor Mutational Burden, T-VEC—Talimogene Laherparepvec, XP—Xeroderma Pigmentosum.

## Data Availability

No new data were created or analyzed in this study. Data sharing is not applicable to this article.
